# Consequences of Lamin B1 and Lamin B Receptor Downregulation in Senescence

**DOI:** 10.3390/cells7020011

**Published:** 2018-02-06

**Authors:** Emilie Lukášová, Aleš Kovařík, Stanislav Kozubek

**Affiliations:** 1 Department of Cell Biology and Radiobiology, Institute of Biophysics, Czech Academy of Sciences, Královopolská 135, Brno 61265, Czech Republic; kozubek@ibp.cz; 2 Department of Molecular Epigenetics, Institute of Biophysics, Czech Academy of Sciences, Královopolská 135, Brno 61265, Czech Republic; kovarik@ibp.cz

**Keywords:** lamin B receptor, lamin B1, lamin A/C, heterochromatin tether, constitutive heterochromatin, cellular senescence, centromere-specific satellite heterochromatin

## Abstract

Anchoring of heterochromatin to the nuclear envelope appears to be an important process ensuring the spatial organization of the chromatin structure and genome function in eukaryotic nuclei. Proteins of the inner nuclear membrane (INM) mediating these interactions are able to recognize lamina-associated heterochromatin domains (termed LAD) and simultaneously bind either lamin A/C or lamin B1. One of these proteins is the lamin B receptor (LBR) that binds lamin B1 and tethers heterochromatin to the INM in embryonic and undifferentiated cells. It is replaced by lamin A/C with specific lamin A/C binding proteins at the beginning of cell differentiation and in differentiated cells. Our functional experiments in cancer cell lines show that heterochromatin in cancer cells is tethered to the INM by LBR, which is downregulated together with lamin B1 at the onset of cell transition to senescence. The downregulation of these proteins in senescent cells leads to the detachment of centromeric repetitive sequences from INM, their relocation to the nucleoplasm, and distension. In cells, the expression of LBR and LB1 is highly coordinated as evidenced by the reduction of both proteins in LBR shRNA lines. The loss of the constitutive heterochromatin structure containing LADs results in changes in chromatin architecture and genome function and can be the reason for the permanent loss of cell proliferation in senescence.

## 1. Function of Lamin B Receptor in Anchoring Chromatin to the Inner Nuclear Membrane

It has been well established that the spatial organization of chromatin plays a critical role in genome functions [1]. The majority of eukaryotic nuclei has a conventional nuclear architecture with euchromatin located predominantly in the internal nucleus, whereas heterochromatin is surrounding the inner side of nuclear envelope and the nucleolus. This functional chromatin arrangement is maintained by means of binding of peripheral heterochromatin sequences to the nuclear envelope (NE). Guelen et al. [2] found that genome–lamina interactions occur in more than 1300 discrete domains, thus dividing the human genome into large sharply demarcated domains of about 0.1–10 Mb in size. These lamina-associated domains (LADs) are characterized by repressive chromatin, showing that nuclear lamina represents a major structural element for the organization of the nuclear genome. Solovei et al. [3] identified the existence of two types of chromatin attachment to lamina: one is executed by means of the lamin B receptor (LBR) in embryonic and non-differentiated cells and the other is mediated by specific lamin A/C binding proteins that are expressed in differentiated cells [4]. Among these the LEM domain proteins (LAP2-emerin-MAN1) carrying a unique globular module of about 40 amino acids are the most prominent ones. LEM-domain proteins present a growing family of nonrelated proteins of the inner nuclear membranes (INMs) [5], linking this membrane and lamin A/C to chromatin during the interphase. LEM-domain proteins share some important properties with LBR: they are anchored to the INM, they interact with lamins, and they bind to chromatin and/or DNA through their binding partners [6]. LBR is a protein of the INM, which preferentially binds lamin B1, and its mutations are known to cause Pelger–Huet anomaly in humans. The Tudor domain of LBR selectively interacts with heterochromatin and represses transcription by binding to chromatin regions marked by specific histone modifications [7,8,9]. It follows from the work of Clowney et al. [10] that LBR and B-type lamins are able to tether heterochromatin to the INM. However, the results of Kim et al. [11] and Yang et al. [12] indicate that B-type lamins can be dispensable in this function because cells from mice lacking both lamin B1 and lamin B2 retain a conventional nuclear architecture in the absence of lamin A/C. This can be due to the presence of LBR that has several transmembrane domains for attachment to the INM [3].

## 2. Distinct Types of Heterochromatin Attachment to the Nuclear Membrane in Undifferentiated (Embryonic) and Differentiated Cells

Heterochromatin tethers support the essential organization of the structure and function of the chromatin [13,14]. Each of the chromatin tethers is responsible for the formation of specific higher order chromatin structure and the regulation of gene expression. Two different types of heterochromatin tethers distinguish cells that are able to proliferate from cells that completed proliferation and are differentiating. While the chromatin structure arranged by the attachment of heterochromatin to the INM by LBR in embryonic and non-differentiated cells allows the active expression of genes that participate in cell proliferation, heterochromatin tethers that are executed by lamin A/C by LEM-domain proteins change this chromatin structure to enable the silencing of proliferative genes while activating the expression of new genes that are specific for different types of differentiated cells. This activation of cell type-specific genes is assured by binding of the heterochromatin to lamin A/C by the LEM-domain proteins, specifically expressed in the specific cell type. Experiments by Solovei et al. [3] have shown that the pattern of LEM-domain protein expression is cell type specific, while none of the LEM-domain proteins seem to be universally expressed in mammalian cells. The LEM-domain proteins cooperate with lamin A/C in tethering peripheral heterochromatin to the INM. Different LEM proteins and their combination mediate heterochromatin binding to lamin A/C, depending on the cell type and developmental stage [15,16,17]. During development and cell differentiation, LBR and lamin A/C expression is sequential and coordinated [3]. Initially, in non-differentiated cells, only LBR is expressed while later at the onset of cell differentiation it is replaced by lamin A/C. Expression of both these proteins in differentiated cells is rarely seen. Experiments in knock-out mice have shown that in most cell types, deletion of lamin A/C is compensated by prolonged expression of LBR [3]. However, no data are available showing the attachment of heterochromatin to lamina in senescent cells.

## 3. Lamin B is Downregulated in Senescence

Cellular senescence is a cellular response to a variety of stresses [18,19,20]. It is accompanied by a set of characteristic morphological and physiological features that distinguish senescent cells not only from proliferating cells but also from quiescent or terminally differentiated cells [19,20]. Because senescence plays an important role in both normal physiology and diverse pathologies, it is important to understand its molecular basis well. Typical features of senescence include irreversible proliferation arrest, enlarged cellular morphology, expression of senescence-associated β-galactosidase activity (SA-β-gal) [20], enhanced nuclear heterochromatinization [19], a senescence-associated secretory phenotype [21,22], and DNA damage signaling [23,24]. These senescence attributes are generally manifested by cells in replicative senescence (due to telomere shortening) [25] and the so-called premature senescence due to different stressors such as activated oncogene, oxidative stress, and other DNA damaging insults [26,27,28].

The onset and maintenance of the senescent state involve the action of two major tumor suppressive pathways, p53-p21 and p16^ink4a^-pRb [29,30]. Upregulation of p16^INK4a^ is particularly prevalent in benign lesions, although is often lost upon malignancy [31], and its expression can be dispensable in senescence [32,33]. Most known senescence-associated markers have been obtained in studies of oncogene-induced senescence or replicative senescence in human diploid fibroblasts [19,34]. They include large senescence-associated heterochromatin foci (SAHF) enriched in heterochromatin markers such as H3K9me3 and HP1 proteins. However, SAHF formation does not occur in all senescent cells [28]. It is absent, for example, in human mammary carcinoma MCF7 and fibrosarcoma U2OS cells in which senescence has been induced by γ-irradiation [33] or by inhibition of replication and Chk1 kinase or ATR kinases.

In recent years, it was reported that lamin B1 (LB1) expression is reduced in replicative and oncogene-induced senescence, which delays cell proliferation and promotes cellular senescence via a p53- and Rb-dependent mechanism [35,36,37,38]. Chandra et al. [39] studied this senescence-associated structure in detail to gain insight into the function of SAHF. Because a key feature of senescent nuclei is the loss of LB1—which correlates with SAHF formation in fibroblasts after senescence induction by oncogene activation—they assumed that this loss might be involved in the architectural changes of chromatin leading to the formation of SAHF [40]. Independently, Sadaie et al. [41] showed that during senescence, LB1 is preferentially depleted from the chromatin regions containing LADs enriched for H3K9me3, a characteristic for constitutive heterochromatin (cHC). The authors supposed that this release of cHC containing LADs from the nuclear membrane would promote the formation of SAHF. In addition, these authors observed that despite its global reduction there was an increased binding of LB1 to gene-rich regions marked with H3K27me3 (a characteristic for facultative heterochromatin). However, there are no known specific reader sequences of LB1 for the recognition of lysine-methylated residues of chromatin. The recognition of these sequences ensures LBR joining with LB1, as found earlier [9,42,43,44]. We observed LB1 foci of variable sizes scattered through the chromatin, but not colocalizing with the INM in senescent nuclei of MCF7 and U2OS cells [33] and assume that these foci might represent protein degradation centers [45].

## 4. The Onset of Senescence is Accompanied by a Coordinated Downregulation of Lamin B Receptor and Lamin B1 Expression

Our results show [33] downregulation of both LBR and LB1 at the onset of senescence induced by γ-irradiation in two cancer cell lines (MCF7 and U2OS). To further explore the apparent coordinated behavior of both proteins in cellular processes we performed experiments: (i) To confirm the capacity of LBR to attach heterochromatin (HC) to the INM in cycling cancer cells and to detach it from the INM after downregulation in senescence. (ii) To establish stable cell lines in which LBR expression was reduced by shRNA constructs [3,7,9,44]. We selected the pericentric satellite heterochromatin (CSH), which represents about 70% of human HC, and followed its attachment to lamina in cancer cells MCF7 and U20S and in these cells transferred to senescence by γ-irradiation. Specifically, the role of LBR was followed in the attachment of CSH in two chromosomes with HC > EC (heterochromatin-rich chromosomes) and two chromosomes with HC < EC (heterochromatin-poor chromosomes). We found a high fraction (>80%) of CSH in heterochromatin-rich (gene-poor) chromosomes colocalizing with the INM in these cancer cells. In heterochromatin-poor chromosomes this was about 50%. More than half of these sequences detached from the INM and relocalized to the nucleoplasm where they were decondensed, not only in the beginning of senescence where LBR and LB1 were lost but also in cells where LBR expression was reduced by LBR-specific shRNA. Colocalization of CSH with the INM also decreased significantly—by approximately two-thirds—in heterochromatin-rich and by one-third in euchromatin-rich chromosomes in cells with shRNA-reduced expression of LBR. These results confirmed the role of LBR as a constitutive heterochromatin tether in proliferating cancer cells [3,9,10]. Moreover, nuclei of senescent cells showed elevated numbers of centromeric signals in different cell lines suggesting that the detachment of centromere-specific satellite heterochromatin from lamina and its decompaction in the nucleoplasm may induce endo-reduplication of this chromatin. This can be related to the results of De Cecco et al. [46] showing that the transcription of satellite DNA, as well as transposable elements, increased during replicative senescence. Our results showing the detachment of CSH of chromosomes with the prevailing amount of heterochromatin (mainly of chromosome 18) from lamina in cells with reduced expression of LBR and LB1 are in consensus with the results of Malhas et al. [47]. They showed that an absence of LB1 or its full-length protein resulted in the relocation of chromosome 18 from the nuclear periphery to the nuclear center in mouse embryonic fibroblasts; this was followed by decondensation of this chromosome and changes in the expression of some of its genes. No changes were observed in chromosome 19, which is located in the center of these cells’ nuclei. Even if the authors did not follow changes in the level of LBR, it could be supposed on the basis of our results, that in the absence of LB1 or in the presence of its incomplete structure the heterochromatin tether may not be executed by LBR in these embryonic cells [3]. This results in relocation of chromosome 18 from the nuclear membrane to the nuclear center.

LBR protein is known to contain specific regions for heterochromatin attachment, recognition of specific histone methylations, HP1γ and, in addition, the attachment of LB1 [7,9,42,43,44,48]. The role of LBR in an un- or early-differentiated state of cells was also demonstrated by Clowney et al. [10] who showed that transgenic expression of LBR deregulates the differentiation of olfactory neurons. The binding of LBR, but not LB1 to heterochromatin, also follows from the stability of LBR associated with condensed chromatin and the INM during the late stage of apoptosis, while lamin B1 is proteolyzed at an early stage [49]. The protease resistance of this LBR association, which plays a major role during apoptosis, is also likely to be important in nuclear membrane reassembly in late anaphase [50]. The functional dependence of LB1 on LBR results from current downregulation of both these proteins at reduced expression of LBR by LBR-specific shRNA shown in our experiments [33]. However, downregulation of both these proteins by LBR shRNA did not induce senescence. Clones of MCF7 and U2OS with reduced expression of these proteins exhibited slower proliferation compared to the parental cells, formed higher numbers of micronuclei (MN) showing higher permeability of the nuclear membrane, and were transferred to senescence by γ-irradiation similarly to their parental cells. Even if it seems that the downregulation of LBR and LB1 is necessary for cell transition to senescence, it does not elicit this process. It is not yet known what gives the impulse to LBR downregulation at the onset of senescence. It is possible that genotoxic stress used in our experiments or other forms of stress inducing senescence cause mutations in the genes participating in LBR expression or the stability of this protein in INM. Our results show that expression of the LBR gene decreased progressively during 72 h post-irradiation (PI). However, the protein disappeared from the nuclear membrane during 24 h PI, indicating reduced stability of the protein at the INM. This might be due to increased phosphorylation [48] or other posttranslational modifications of this protein due to stress-induced activity of specific kinases or mutations in genes of proteins that help to stabilize the position of LBR in the INM. It is well known that LBR becomes hyperphosphorylated at the beginning of mitosis, which results in its dissociation from the INM and disassembly of the nuclear envelope [51,52]. We observed that some cells, especially the U2OS cell line, excrete LBR protein into the cytoplasm in a dispersed form and or bound to heterochromatin fragments. We assume that this LBR and chromatin fragments are degraded by autophagy machinery that is frequent in senescent cells and that serve for recycling the cellular constituents and cell remodeling [53]. The attachment of heterochromatin to the INM by means of LBR and LB1 establishes conditions for active transcription of genes responsible for cell proliferation in undifferentiated, cycling cells [3,9].

It is very interesting that in undifferentiated and differentiated cells the higher order chromatin structure is set by the anchoring of heterochromatin to the INM by means of distinct proteins of the INM that simultaneously binds to one of the lamins: lamin B receptor binds to lamin B1 in undifferentiated and embryonic cells and different LEM-domain proteins and other transmembrane mediators bind to lamin A/C in various differentiated cells [6]. This shows that lamins bind to heterochromatin indirectly but have a very important role in anchoring heterochromatin to the INM, which is not yet fully clarified. The experiments of Solovei et al. [3] show that the expression of LEM-domain proteins is cell type and developmental stage specific. None of the LEM-domain proteins are present in any cell type lacking LBR. Therefore, no individual LEM-domain protein could be the universal mediator of the lamin A/C-dependent peripheral heterochromatin tether. Processes of cell differentiation directed by a heterochromatin tether with the participation of lamin A/C are very complicated and participation of the different proteins in this process is not yet completely clear.

## 5. Consequences of LBR Downregulation in Senescence

Genome-wide mapping of LB1 has identified lamina-associated domains (LADs) that are gene poor and guanine–cytosine poor, enriched for histone repressive marks [2,54] and repetitive sequences LINE, and are specific for heterochromatin. LAD domains in which heterochromatin is anchored to lamina and the INM by LBR in undifferentiated cells or LEM-domain proteins in differentiated cells are distributed along chromosomes and cover about 35% of the genome [2,54,55,56]. Pericentromeric satellite heterochromatin CSH can be considered as immense LADs, specific for each chromosome, invariably found at the nuclear or nucleolar peripheries [54,57,58,59,60].

**Figure 1:**
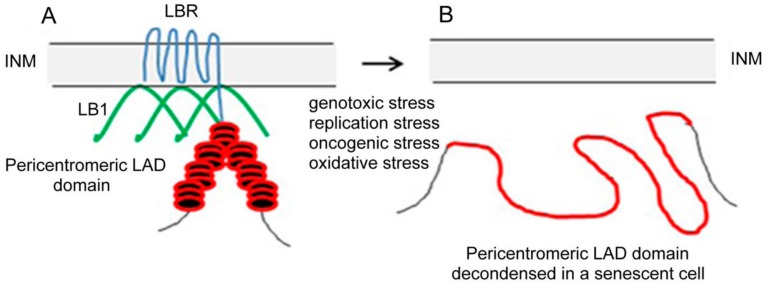
Schematic representation of a pericentric heterochromatin LAD domain (red) and lamin B1 (green) anchorage to INM (grey) by means of LBR (blue) in an interphase nucleus of a cancer cell (**A**) and distended pericentric heterochromatin LAD domain in a senescent nucleus (**B**). In (B) the LAD domain of a pericentric heterochromatin is no longer attached to INM because LBR that anchored this condensed domain to INM in interphase nucleus was lost together with LB1 after cell transition to senescence. The loss of both proteins in senescence leads to the relocation of pericentric heterochromatin from INM to the nucleoplasm and to its distension.

The downregulation of LBR and LB1 in the beginning of senescence induces several consecutive changes that eventually lead to irreversible alterations in nuclear architecture. The evidence for this hypothesis comes from several experimental systems. In irradiated cancer cells, we observed detachment of peri/centromeric satellite heterochromatin (enriched in the epigenetic H3K9me3 mark) from the inner nuclear membrane and its relocation to the nucleoplasm [33]. Interestingly, the heterochromatin-rich chromosomes seem to be more affected than those that are heterochromatin poor. This indicates that chromosomes containing more heterochromatin are more tightly attached to the INM due to the higher number of LADs in which chromatin is anchored to the INM by LBR and LB1. Further, high-resolution confocal microscopy has shown marked distension of peri/centromeric heterochromatin following their relocation from the inner nuclear membrane to the nucleoplasm (Figure 1). Using specific probes for peri/centromeric repetitive sequences of specific chromosomes, we were able to follow changes in their structure and nuclear location; this was not possible for the rest of the LADs of different sizes [2] scattered along the genome. However, owing to the loss of the principal tether protein LBR/LB1 in the beginning of cancer cell senescence, we can speculate that the other heterochromatic loci containing LADs that are specifically recognized by LBR were also detached from the lamina and unfolded. For this assumption, it attests higher changes in the location and distension of CHS of chromosomes with higher content of HC > EC compared to CSH of chromosomes containing HC < EC. Significantly, a similar distension of peri/centromeric satellite sequences in human fibroblasts induced to senescence by diverse stresses (termed by authors “senescence-associated distension of satellite (SADS)”) was also reported by Swanson et al. [61]. These observations made in independent systems indicate that the phenomenon of satellite heterochromatin decompaction is common in senescence. De Cecco et al. [46], analyzing nucleosome densities in nuclei of senescent cells, showed that repetitive sequences and pericentric constitutive heterochromatin became relatively more open (decondensed), while open regions became generally more closed (condensed) with the exception of some specific genes; this indicates that the density of nucleosomes become more homogenous. Sadaie et al. [41] were the first to implicate the dynamic changes in LB1 for SHAF formation and gene regulation during senescence of human diploid fibroblasts. These findings of spatial chromatin changes in senescent cells with reduced LB1 were confirmed by a comprehensive study using Hi-C by Chandra et al. [39]. Both of these excellent studies found a connection between the reduction of LB1 and rearrangement of the genome architecture during cellular senescence, consisting of changes in constitutive heterochromatin density and location. However, they were not interested in the reasons for this genome rearrangement in senescence or why this rearrangement is connected with the reduction of LB1. It is conceivable that these changes in chromatin configuration may induce a number of changes in gene expression. We propose that the trigger of chromatin alterations in senescent cells could primary be the loss of the heterochromatin anchoring protein to the INM, the LBR. While the heterochromatin tether executed in embryonic and undifferentiated cells by LBR is replaced by lamin A/C with specific LEM-domain proteins after downregulation of this protein at the beginning of cell differentiation, there is no known heterochromatin tether for replacing the downregulated LBR after cells transition to senescence. The cause may be just in the loss of constitutive heterochromatin structure. Heterochromatin tethers cannot be restored in senescent cells that have lost constitutive heterochromatin and LAD-domain structure. The loss of constitutive heterochromatin structure results in profound changes in chromatin architecture and its function. Decompaction of heterochromatin in LAD domains renders these domains non-functional, preventing attachment to the INM and nuclear lamina. We speculate that loss of function of the LAD domain might be an irreversible step leading to the permanent cessation of cell proliferation.

## 6. Conclusions

The results of further experiments will show whether our conclusions regarding the fate of senescent cells after the loss of constitutive heterochromatin structure are real or not and whether they could be found conditions for this structure restoration.

## References

[1] Woodcock C.L., Ghosh R.P. (2010). Chromatin higher order structure and dynamics. Cold Spring Harb. Perspect. Biol..

[2] Guelen L., Pagie L., Brasset E., Meuleman W., Faza M.B., Talhout W., Eussen B.H., de Klein A., Wessels L., de Laat W. (2008). Domain organization of human chromosomes revealed by mapping of nuclear lamina interactions in single human cells. Nature.

[3] Solovei I., Wang A.S., Thanisch K., Schmidt C.S., Krebs S., Zwerger M., Cohen T.V., Devys D., Foisner R., Peichl L. (2013). LBR and lamin A/C sequentially tether peripheral heterochromatin and inversely regulate differentiation. Cell.

[4] Andrés V., González J.M. (2009). Role of A-type lamins in signaling, transcription, and chromatin organization. J. Cell Biol..

[5] Wagner N., Krohne G. (2007). LEM-Domain proteins: New insights into lamin-interacting proteins. Int. Rev. Cytol..

[6] Brachner A., Foisner R. (2011). Evolvement of LEM proteins as chromatin tethers at the nuclear periphery. Biochem. Soc. Trans..

[7] Makatsori D., Kourmouli N., Polioudaki H., Shultz L.D., McLean K., Theodoropoulos P.A., Singh P.B., Georgatos S.D. (2004). The inner nuclear membrane protein lamin B receptor forms distinct microdomains and links epigenetically marked chromatin to the nuclear envelope. J. Biol. Chem..

[8] Olins A.L., Rhodes G., Welch D.B., Zwerger M., Olins D.E. (2010). Lamin B receptor: Multi-tasking at the nuclear envelope. Nucleus.

[9] Hirano Y., Hizume K., Kimura H., Takeyasu K., Haraguchi T., Hiraoka Y. (2012). Lamin B receptor recognizes specific modifications of histone H4 in heterochromatin formation. J. Biol. Chem..

[10] Clowney E.J., LeGros M.A., Mosley C.P., Clowney F.G., Markenskoff-Papadimitriou E.C., Myllys M., Barnea G., Larabell C.A., Lomvardas S. (2012). Nuclear aggregation of olfactory receptor genes governs their monogenic expression. Cell.

[11] Kim Y., Sharov A.A., McDole K., Cheng M., Hao A., Fan C.M., Giano M.N., Ko M.S., Zheng Y. (2011). Mouse B-type lamins are required for proper organogenesis but not by embryonic stem cells. Science.

[12] Yang S.H., Chang S.Y., Yin L., Tu Y., Hu Y., Yoshinaga Y., de Jong P.J., Fong L.G., Young S.G. (2011). An absence of both lamin B1 and lamin B2 in keratinocytes has no effect on cell proliferation or the development of skin and hair. Hum. Mol. Genet..

[13] Broers J.L., Ramaekers F.C., Bonne G., Yaou R.B., Hutchison C.J. (2006). Nuclear lamins: Laminopathies and their role in premature ageing. Phisiol. Rev..

[14] Holmer L., Worman H.J. (2001). Inner nuclear membrane proteins: Functions and targeting. Cell. Mol. Life Sci..

[15] Ikegami K., Egelhofer T.A., Strome S., Lieb J.D. (2010). Caenorhabditis chromosome arms are anchored to nuclear membrane via discontinuous association with LEM-2. Genome Biol..

[16] Mattout A., Pike B.L., Towbin B.D., Bank E.M., Gonzales-Sandoval A., Stadier M.B., Meister P., Gruenbaum Y., Gasser S.M. (2011). An EDMD mutation in *C. elegans* lamin blocks muscle-specific gene relocation and compromises muscle integrity. Curr. Biol..

[17] Towbin B.D., Gonzáles-Aguilera C., Sack R., Gaidatzis D., Kalck V., Meister P., Askjaer P., Gasser S.M. (2012). Step-wise methylation of histone H3K9 positions heterochromatin at the nuclear periphery. Cell.

[18] Campisi J., d’Adda di Fagagna F. (2007). Cellular senescence: When bad things happen to good cells. Nat. Rev. Mol. Cell Biol..

[19] Narita M., Nunez S., Heard E., Narita M., Lin A.W., Hearn S.A., Spector D.L., Hannon G.J., Lowe S.W. (2003). Rb-mediated heterochromatin formation and silencing of E2F target genes during cellular senescence. Cell.

[20] Dimri G.P., Lee X., Basile G., Acosta M., Scott G., Roskelley C., Medrano E.E., Linskens M., Rubelj I., Pereira-Smith O. (1995). A biomarker that identifies senescent human cells in culture and in aging skin in vivo. Proc. Natl. Acad. Sci. USA.

[21] Kuilman T., Michaloglou C., Vredeveld L.C., Douma S., van Doorn R., Desmet C.J., Aarden L.A., Mooi W.J., Peeper D.S. (2008). Oncogene-induced senescence relayed by an interleukin-dependent inflammatory network. Cell.

[22] Novakova Z., Hubackova S., Kosar M., Janderova-Rossmeislova L., Dobrovolna J., Vasicova P., Vancurova M., Horejsi Z., Hozak P., Bartek J. (2010). Cytokine expression and signaling in drug-induced cellular senescence. Oncogene.

[23] d’Adda di Fagagna F., Reaper P.M., Clay-Farrace L., Fiegler H., Carr P., Von Zglinicki T., Saretzki G., Carter N.P., Jackson S.P. (2003). A DNA damage checkpoint response in telomere-initiated senescence. Nature.

[24] Von Zglinicki T., Saretzki G., Ladhoff J., d’Adda di Fagagna F., Jackson S.P. (2005). Human cell senescence as a DNA damage response. Mech. Ageing Dev..

[25] Sedivy J.M. (2007). Telomeres limit cancer growth by inducing senescence: Long-sought in vivo evidence obtained. Cancer Cell.

[26] Von Zglinicki T. (2002). Oxidative stress shortens telomeres. Trends Biochem. Sci..

[27] Serrano M., Lin A.W., McCurrach M.E., Beach D., Lowe S.W. (1997). Oncogenic ras provokes premature cell senescence associated with accumulation of p53 and p16^INK4a^. Cell.

[28] Kosar M., Bartkova J., Hubackova S., Hodny Z., Lukas J., Bartek J. (2011). Senescence-asociated heterochromatin foci are dispensable for cellular senescence, occur in a cell type- and insult-dependent manner and follow expression of p16^ink4a^. Cell Cycle.

[29] Chen Q.M., Bartholomew J.C., Campisi J., Acosta M., Reagan J.D., Ames B.N. (1998). Molecular analysis of H_2_O_2_-induced senescent-like growth arrest in normal human fibroblasts: P53 and Rb control G1 arrest but not cellreplication. Biochem. J..

[30] Beausejour C.M., Krtolica A., Galimi F., Narita M., Lowe S.W., Yaswen P., Campisi J. (2003). Reversal of human cellular senescence: Roles of the p53 and p16 pathways. EMBO J..

[31] Michaloglou C., Vredeveld L.C.W., Soengas M.S., Denoyelle C., Kuilman T., van der Horst C.M., Majoor D.M., Shay J.W., Mooi W.J., Peeper D.S. (2005). BRAF^E600^-associated senescence-like cell cycle arrest of human naevi. Nature.

[32] Bartkova J., Rezaei N., Lintos M., Karakaidos P., Kletsas D., Issaeva N., Vassiliou L.F., Kolettas E., Niforou K., Zoumpourlis V.C. (2006). Oncogene-induced senescence is part of the tumorigenesis barrier imposed by DNA damage checkpoints. Nature.

[33] Lukášová E., Kovařík A., Bačíková A., Falk M., Kozubek S. (2017). Loss of lamin B receptor is necessary to induce cellular senescence. Biochem. J..

[34] Zhang R., Chen W., Adams P.D. (2007). Molecular dissection of formation of senescence-associated heterochromati foci. Mol. Cell. Biol..

[35] Shimi T., Butin-Israeli V., Adam S.A., Hamanaka R.B., Goldman A.E., Lucas C.A., Shumaker D.K., Kosak S.T., Chandel N.S., Goldman R.D. (2011). The role of lamin B1 in cell proliferation and senescence. Genes Dev..

[36] Freund A., Laberge R.M., Demaria M., Campisi J. (2012). Lamin B1 loss is a senescence-associated biomarker. Mol. Biol. Cell.

[37] Dreesen O., Chojnowski A., Ong P.F., Zhao T.Y., Common J.E., Lunny D., Lane E.B., Lee S.J., Vardy L.A., Stewart C.L. (2013). Lamin B1 fluctuations have differential effects on cellular proliferation and senescence. J. Cell Biol..

[38] Shah P.P., Donahue G., Otte G.L., Capell B.C., Nelson D.M., Cao K., Aggarwala V., Cruickshanks H.A., Rai T.S., McBryan T. (2013). Lamin B1 depletion in senescence cells triggers large-scale changes in gene expression and the chromatin landscape. Genes Dev..

[39] Chandra T., Ewels P.A., Schoenfelder S., Furlan-Magaril M., Wingett S.W., Kirschner K., Thuret J.-Y., Andrews S., Fraser P., Reik W. (2015). Global reorganization of the nuclear landscape in senescence cells. Cell Rep..

[40] Chandra T., Kirschner K., Thuret J.-Y., Pope B.J., Ryba T., Newman S., Ahmed K., Samarajiwa S.A., Salama R., Carroll T. (2012). Independence of repressive histone markers and chromatin compaction during senescent heterochromatic layer formation. Mol. Cell.

[41] Sadaie M., Salama R., Carroll T., Tomimatsu K., Chandra T., Young A.R.J., Narita M., Pérez-Mancera P.A., Bennett D.C., Chong H. (2015). Redistribution of the lamin B1 genomic binding profile affects rearrangement of heterochromatic domains and SAHF formation during senescence. Genes Dev..

[42] Ye Q., Worman H.J. (1994). Primary structure analysis and lamin B and DNA binding of human LBR, an integral protein of the nuclear envelope inner membrane. J. Biol. Chem..

[43] Worman H.J., Yuan J., Blobel G., Georgatos S.P. (1988). A lamin B receptor in the nuclear envelope. Proc. Natl. Acad. Sci. USA.

[44] Ye Q., Worman H.J. (1996). Interaction between an integral protein of the nuclear envelope inner membrane and human chromodomain proteins to Drosophilla HP1. J. Biol. Chem..

[45] Von Mikecz A., Chen M., Rockel T., Scharf A. (2008). The nuclear ubiquitin-proteasome system: Visualization of proteasomes, protein aggregates, and proteolysis in the cell nucleus. Nucleus.

[46] De Cecco M., Criscione S.W., Peckham E.J., Hillenmeyer S., Hamm E.A., Manivannan J., Peterson A.L., Kreiling J.A., Neretti N., Sedivy J.M. (2013). Genomes of replicative senescent cells undergo global epigenetic changes leading to gene silencing and activation of transposable elements. Aging Cell.

[47] Malhas A., Lee C.F., Sanders R., Sounders N.J., Vaux D.J. (2007). Defects in lamin B1 expression or processing affect interphase chromosome position and gene expression. J. Cell Biol..

[48] Nikolakaki E., Meier J., Simons G., Georgatos S.D., Giannakouros T. (1997). Mitotic phosphorylation of the lamin B receptor by a serine/arginine kinase and p34(cdc2). J. Biol. Chem..

[49] Duband-Goulet I., Courvalin J.-C., Buendia B. (1998). LBR, a chromatin and lamin binding protein from the inner nuclear membrane, is proteolysed at late stage of apoptosis. J. Cell Sci..

[50] Ellenberg J., Siggia E.D., Moreira J.E., Smith C.L., Presley J.F., Worman H.J., Lippincott-Schwartz J. (1997). Nuclear membrane dynamics and reassembly in living cells: Targeting of an inner nuclear membrane protein in interphase and mitosis. J. Cell Biol..

[51] Nikolakaki E., Milonis I., Giannakouros T. (2017). Lamin B receptor: Interplay between structure, function and localization. Cells.

[52] Tseng L.C., Chen R.H. (2011). Temporal control of nuclear envelope assembly by phosphorylation of lamin B receptor. Mol. Biol. Cell.

[53] Ivanov A., Pawlowski J., Manoharan I., van Tuyn J., Nelson D.M., Rai T.S., Shah P.S., Hewitt G., Korolchuk V.I., Passos J.F. (2012). Lysozome-mediated processing of chromatin in senescence. J. Cell Biol..

[54] Peric-Hupkes D., Meuleman W., Pagie L., Bruggeman S.W., Solovei I., Brugman W., Graf S., Flicek P., Kerkhoven R.M., Reinders M. (2010). Molecular maps of the reorganization of genom-nuclear lamina interactions during differentiation. Mol. Cell.

[55] Meulman W., Peric-Hupkes D., Kind J., Beaudry J.B., Pagie L., Kellis M., Reinders M., Wessels L., van Steensel B. (2013). Constitutive nuclear lamina-genomeinteractions are highly conserved and associated with A/T-rich sequence. Genome Res..

[56] Solovei I., Thanisch K., Feodorova Y. (2016). How to rule the nucleus: *Divide et impera*. Curr. Opin. Cell Biol..

[57] Wijchers P.J., Geeven G., Eyres M., Bergsma A.J., Janssen M., Versteegen M., Zhu Y., Schell Y., Vermeulen C., de Vit E. (2015). Characterization and dynamics of pericentromere-associated domains in mice. Genome Res..

[58] Solovei I., Schemelleh L., During K., Engelhardt A., Stein S., Cremer C., Cremer T. (2004). Differences in centromere positioning of cycling and postmitotic human cell types. Chromosoma.

[59] Bouwman B.A., de Laat W. (2015). Getting the genome in shape: The formation of loops, domains and compartments. Genome Biol..

[60] Weierich C., Brero A., Stein S., von Hase J., Cremer C., Cremer T., Solovei I. (2003). Three-dimensional arrangements of centromeres and telomeres in nuclei of human and murine lymphocytes. Chromosom. Res..

[61] Swanson E.C., Manning B., Zhang H., Lawrence J.B. (2013). Higher-order unfolding of satellite heterochromatin is a consistent and early event in cell senescence. J. Cell Biol..

